# Fungi Follow Flora, Bacteria Track the Seasons: A Tale of a Changing Landscape

**DOI:** 10.1007/s00248-025-02568-3

**Published:** 2025-06-20

**Authors:** Emily L. Embury, Adriana L. Romero-Olivares

**Affiliations:** Department of Biology, New Mexico State University, Las Cruces, NM USA

**Keywords:** Fungi, Bacteria, Shrub encroachment, Dryland, Landscape

## Abstract

Microbes play critical roles in dryland ecosystems, driving nutrient cycling, soil stability, and plant interactions. Despite their ecological importance, few studies have examined how microbial communities respond to vegetation changes in arid landscapes. In the northern extent of the Chihuahuan Desert, the encroachment of woody shrubs into grasslands has been occurring since the 1800s, largely driven by extensive livestock grazing and increased drought levels. In this study, we investigated how microbial communities respond to both biotic (i.e., vegetation) and abiotic (i.e., seasonality) factors, how they assemble in a changing landscape, and which taxa may be particularly responsive to shrub encroachment or even facilitating this transformation. We assessed microbial communities using soil surface samples across five distinct seasonal periods in a grassland-to-shrubland gradient in the Jornada Experimental Range in the Chihuahuan Desert through the use of phospholipid fatty-acid analysis and DNA metabarcoding techniques. Our findings reveal that bacterial and fungal biomass are significantly influenced by seasonal changes, with strong correlations to humidity and temperature fluctuations. We also found that fungal community assembly and diversity were highly impacted by vegetation whereas seasons were more impactful on bacteria. Our results support the idea that microbes may be playing a crucial role in facilitating the grassland-to-shrubland transition. Overall, our study highlights the complex interactions between microbial communities and biotic and abiotic factors in dryland systems. These findings are essential for understanding the future of dryland ecosystems undergoing shrub encroachment and provide a critical foundation for guiding restoration efforts, particularly those looking to incorporate microbial-mediated solutions.

**Supplementary Information:**

The online version contains supplementary material available at 10.1007/s00248-025-02568-3.

## Introduction

Shrub encroachment is a widespread phenomenon affecting dryland ecosystems around the world which cover over 41% of Earth’s terrestrial surface and are expected to expand under future climate change scenarios [1, 2]. Driven by factors such as livestock overgrazing, small mammal activity, and drought [3], this encroachment is altering local temperature patterns [4], reducing biodiversity [5, 6], and disrupting carbon dynamics [7]. Despite the significance of this issue, dryland ecosystems remain understudied, with only a small fraction of ecological research focusing on them [8]. Even more overlooked are the microbial communities in these environments, despite their crucial role in mediating biogeochemical cycles and their interactions with plants, animals, and other microbes [9, 10]. A critical knowledge gap exists regarding the response of microbes and/or their potential role in driving shrub encroachment.

In the northern extent of the Chihuahuan Desert, the largest desert in North America, historically grass-dominated sites included species such as black grama (*Bouteloua eriopoda*), mesa dropseed (*Sporobolus flexuosus*), tobosa (*Hilaria mutica*), and burrograss (*Scleropogon brevifolius*). These sites are now mostly dominated by shrubs like creosote (*Larrea tridentata*), tarbush (*Flourensia cernua*), and honey mesquite (*Prosopis glandulosa*) [11]. Both grasses and shrubs can host microbes within all vegetative structures in addition to forming external relationships with microbes. For example, black grama grass hosts endophytes which benefit its survival [12, 13] while mesquite hosts rhizobia bacteria which provide nitrogen to the plant [14]. Moreover, black grama grass and mesquite shrubs, are both colonized by arbuscular mycorrhizal (AM) fungi [15, 16] which aid in the acquisition of nutrients and abiotic stress [17]. However, not all microbial interactions are beneficial. Both fungi and bacteria can infect plants, obtaining beneficial nutrients while causing various diseases and death [18, 19].

Interactions between microbes and the ecosystem also influence plant communities. For example, microbial functions can change (e.g., saprotrophic to pathogenic or vice versa) within a time span of days to weeks due to environmental conditions such as soil temperature and moisture levels [20]. Beyond just daily or weekly variability, seasonal variability can also drive changes in microbial community compositions and functions [20]. For instance, seasonal variability in the northern extent of the Chihuahuan Desert impacted the abundance of soil bacteria and AM fungal functions across seasons and years of differing precipitation patterns [21]. Variation in microbial functions and abundance are linked to both abiotic and biotic seasonal pressures. In grass systems, biotic factors such the physiological changes of grass roots in the summer are influential on AM fungi functionality. In contrast, soil bacteria are more heavily influenced by abiotic factors such as soil moisture, leading to seasonal declines in gram positive bacterial abundance [21]. The effects of biotic and abiotic factors on microbial communities are also impacted by spatial patterns. For example, during seasonal monsoon rains in the northern extent of the Chihuahuan Desert, undershrub resource islands maintain relatively higher levels of nutrients and microbial biomass in contrast to interspace soils. Additionally, certain microbial enzymes vary substantially between under-shrub and interspace soils [22].

It is clear that investigating microbes can increase understanding of ecosystem functions. Yet, microbial communities in dryland systems are understudied, including in the Chihuahuan Desert. As of 2024, a *Web of Science* search with the keywords “microbial” and “Chihuahuan” only returns 122 results. When the term “encroachment” is added, the search only returns eight results. While a search like this is likely to exclude some relevant papers, it does demonstrate just how few studies exist on Chihuahuan Desert soil microbes in the context of shrub encroachment. However, such a large area of land (~ 174,472 km^2^ in the USA alone) has substantial potential for sequestering carbon and mitigating climate change effects, but climate models are not always well tailored to arid systems and therefore produce uncertain predictions [23]. As microbes are incredibly important in nutrient cycling, integrating information about microbial processes in general, and specifically in changing landscapes of dryland ecosystems, into climate models can help improve model predictions [24].

In this study, we aimed to investigate the response of microbes and/or their potential role in shrub encroachment over space and time. To do this, we assessed the soil bacteria and fungi in a grassland-to-shrubland gradient over a ten-month time span in the Jornada Experimental Range located in the northern extent of the Chihuahuan Desert. We asked (1) how does the microbial community respond in space and time and when considering biotic (i.e., vegetation) and abiotic (i.e., seasonality) factors? (2) how do microbial networks assemble in a grassland-to-shrubland gradient, and (3) which microbial taxa may be responding strongly to shrub encroachment and/or playing a crucial role in facilitating this phenomenon in the northern extent of the Chihuahuan Desert? We hypothesized that the microbial community would respond differently in space and time; specifically, that bacteria would be more responsive to changes in abiotic factors while fungi would be more responsive to changes in biotic factors. And these responses would be reflected in the diversity of the community and its microbial networks and abundance dynamics.

## Methods

### Location

Our study was conducted in the northern region of the Chihuahuan Desert in the Jornada Experimental Range (Jornada), specifically in the Long-Term Ecological Research (LTER) site (32°30′N, 106°47′W, 1188 m above sea level), located in Las Cruces, NM, USA. It is a dryland system with a mean annual precipitation of 230 mm with most of the precipitation events occurring in monsoon-like events from mid-summer to early-fall. The average maximum temperature is 36 °C, usually occurring in the early summer (June), and the average minimum temperature is 13 °C, usually occurring in early winter (January) [25]. The Jornada has historically been dominated by grasslands but has transitioned into a shrubland-dominant state [26]. Honey mesquite is the dominant shrub of the Jornada and has replaced a large portion of black grama grasslands [26, 27].

### Sampling Sites and Plot Setup

**Fig. 1:**
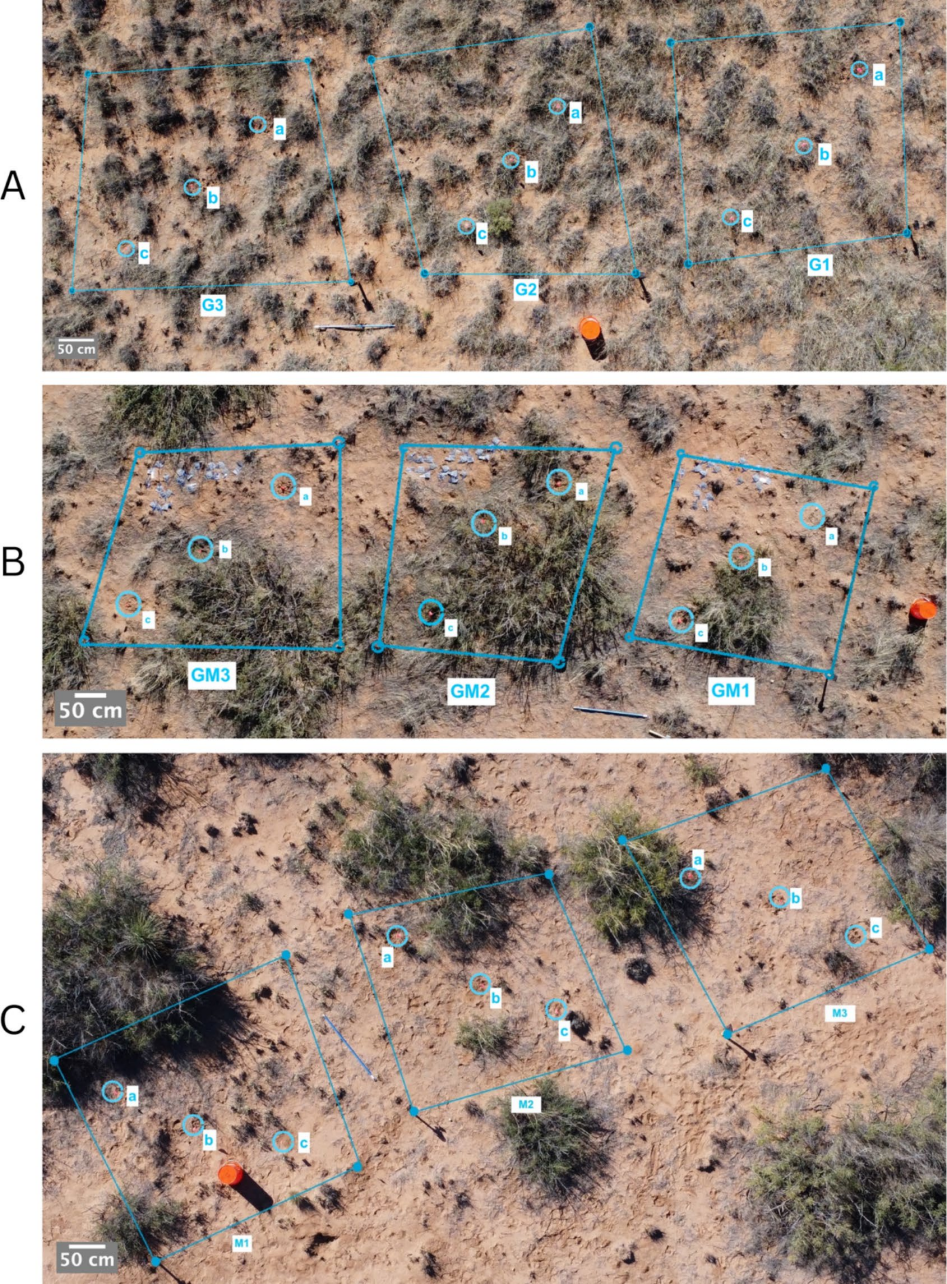
**A** Study plots (labeled G1-G3) in the black grama-dominated vegetation zone. Points labeled a-c indicate the diagonal sampling transect; “G” indicates “grass.” **B** Study plots (labeled GM1-GM3) in the ecotone vegetation zone. Points labeled a-c indicate the diagonal sampling transect; “GM” indicates “grass-mesquite.” **C** Study plots (labeled M1-M3) in the mesquite vegetation zone. Points labeled a–c indicate the diagonal sampling transect; “M” indicates “mesquite”

To assess soil microbes in a grassland-to-shrubland gradient, we identified study sites composed of predominantly black grama (hereafter referred to as the grass site, [32°33′48″N 106°47′54″W]) (Fig. 1A), predominantly mesquite (hereafter referred to as the mesquite site, [32°34′39″N 106°46′57″W]) (Fig. 1C), and a transition zone of both mesquite and black grama (hereafter referred to as the ecotone [32°34′14″N 106°48′13″W]) (Fig. 1B). The study sites were located in the southwestern region of the Jornada with less than 3.5 km of distance between each study site. In each vegetation type, we established three 3 × 3 m plots with 1 m of spacing between each plot. Plot locations were randomly selected within the selected vegetation types. To obtain a good representation of the microbial community across plots, within each individual plot, we selected three sampling points across a diagonal transect; spacing between sampling points was made as equal as possible while still ensuring the plants were not disturbed (Fig. 1).

### Soil Sampling

We sampled during five distinct seasonal periods: October 2022, January 2023, March 2023, May 2023, and July 2023. We selected sampling periods based on distinct temperature and humidity trends observed in collected data from the year prior (Supplementary Fig. 1). These five sampling periods capture all possible combinations of temperature and humidity. That is, high temperature and high humidity, low temperature and low humidity, high temperature and low humidity, and low temperature and high humidity. We collected soil samples along a diagonal transect in each selected plot (*n* = 3). Samples were constrained to the top 2.5 cm of soil. At each sampling point, we collected five samples: one for DNA extraction, two for soil analyses, and two backup samples. With this, we took 15 samples at each plot for a total of 45 samples for each vegetation type. This collection was repeated during each sampling time point. We avoided sampling the same spots by flagging sampling spots. Then, in future samplings, we sampled in parallel to flagged spots keeping the randomized transect approach. Our samples were placed in a portable cooler until they were transported to the laboratory within 10 h. Samples for DNA extraction and soil analyses were stored at − 20 °C and samples for PLFA were stored at − 80 °C until processed within a few months of collection (i.e., 1–8 months depending on collection date).

### Vegetation Cover

We quantified the percentage of vegetation using drone images (Supplementary Fig. 2). Specifically, images of each vegetation type were taken with a DJI mini 2 (Shenzhen DJI Sciences and Technologies Ltd.) flown above each site; all three plots per site were captured in a single photo. To compare photos, we placed a bucket in the frame of each photo for scale. We took images of the sites during each sampling date, totaling five photos of each site. Photos were analyzed using ImageJ (version 1.53t) [28]. We used the “Set Scale” function with the circumference of the bucket in each photo as scale and converted the pixel distance to centimeters. We measured the area of vegetation in centimeters in each site’s plots from each sampling date. Then, averaged to limit errors occurring from differing lighting and angles in the photos that could result in varying measurements.

### Temperature, Humidity, and Precipitation

We obtained data on air temperature, relative humidity, and precipitation from Jornada meteorological stations adjacent to the research sites. All three of the sites in this study were within 200 m of the meteorological stations. The grass plots were adjacent to the Cross-scale Interactions Study (CSIS) Block 8 station [29], the ecotone plots were adjacent to the CSIS Block 7 station [30], and the mesquite plots were adjacent to the CSIS Block 11 station [31]. As these databases go back to 2013, the data was reduced to only October 2022–July 2023 for the purposes of this analysis.

### Environmental Measures

We sent our soil samples to the Regenerative Agricultural (RegenAg) Laboratory in Pleasanton, Nebraska, USA, to measure the ratio of total carbon to nitrogen (C:N) in the soil, soil pH, and microbial biomass in the soil through phospholipid fatty-acid analysis (PLFA).

### DNA Extraction, Sequencing, and Bioinformatics

DNA was extracted from the soil using the QIAGEN DNeasy PowerSoil Pro Kit (QIAGEN Group, Hilden, Germany) following standard procedures. We used a standardized metabarcoding procedure to amplify the ITS2 fungal ribosomal region and construct a DNA library [32]. The fungal ITS2 library was shipped overnight on dry ice to the University of Minnesota Genomics Center for Illumina MiSeq Sequencing. Additionally, DNA samples were sent to the University of Minnesota Genomics Center for 16S library construction and Illumina MiSeq Sequencing.

16S and ITS2 sequences were demultiplexed by the University of Minnesota Genomics Center. After receiving the demultiplexed sequences, we processed them using the DADA2 1.16 pipeline with default parameters on the New Mexico State University Discovery Cluster RStudio Module (R version 4.2.3) [33]. We removed possible contaminants from the ASV tables based on sequences in the control samples using the R package decontam [34]. Using the “isContaminant” function and a threshold of 0.5, 9 contaminants were identified in the bacterial ASV table and were removed. The same procedure was used for the fungal sequences and no substantial contaminants were identified. Demultiplexed sequences and metadata were deposited in NCBI (accession numbers: PRJNA1242850 and PRJNA1242890).

We assigned taxonomy using the SILVA database version 138.1 for bacterial assignments and UNITE database version (release date 7–18–2023) for fungal assignments [35–37]. Any unknowns (0.17% of sequences), eukaryotes (0.01% of sequences), or archaea (0.81% of sequences) were removed from the bacterial dataset. Only taxa assigned to the kingdom Fungi remained after the DADA2 pipeline, so no sequences were removed from the fungal dataset. Sample 93 (an ecotone site sample from May) was removed from the bacterial dataset as there was an abnormally low number of sequences remaining after the DADA2 pipeline.

### Statistics and Community Analyses

All statistical and community analyses were conducted with R (version 4.3.1) [38]. For all statistics, we set the alpha value to 0.05.

#### Phospholipid Fatty-Acid Analysis (PLFA)

We visualized the PLFA data using ggplot2 [39] and separated by vegetation type and month for both bacterial and fungal biomass. To assess significance, we conducted mixed effects models with the percentage of biomass as the dependent variable, the vegetation and month of sampling as the fixed effects, and plot as the random effect. All mixed effects models were conducted using the “lmer” function in the lme4 package (version 1.1–37) [40]. We assessed mixed effects model residuals to ensure normality and homoscedasticity assumptions were met. We ran the Satterthwaite method using the lmerTest package [41] to obtain *p*-values. We conducted Tukey HSD post-hoc analysis to identify specific significant pairwise differences using the emmeans package (version 1.8.9) [42]. We utilized the “cor.test” function in the ggpubr package (version 0.6.0) to assess Pearson correlations between biomass measures and environmental variables [43]. Although our dataset includes repeated measures, our primary goal here was to assess general trends rather than make inference at the individual timepoint level by vegetation. We acknowledge the lack of independence among observations and interpreted these results accordingly.

#### Alpha Diversity

We calculated Shannon-Weiner and Simpson diversity indices to assess variability in species evenness and richness across vegetation types and sampling months. To assess alpha metrics, we used the R packages Vegan (version 2.6–4) and Phyloseq (version 1.46.0) [44, 45]. Prior to calculating alpha diversity, we rarefied the ASV tables [46] for more accurate community comparisons. To rarefy, the lowest sequencing depth was identified and the “rrarefy” function of Vegan [45] was utilized to adjust all sequencing depths to match the lowest depth.

After rarefying the dataset, we calculated alpha diversity using the “estimate_richness” function in Phyloseq [44]. The Shannon-Weiner diversity metric and the Simpson diversity metric were selected for alpha diversity analysis. Both the Shannon-Weiner and Simpson indices estimate species richness and evenness, but Shannon-Weiner is more influenced by species richness whereas Simpson is more influenced by species evenness [47]. After calculating the diversity indices, we assessed the statistical significance of the results using mixed effects model with Satterthwaite method where the diversity index was treated as the dependent variable, the vegetation type and the sampling month were used as the fixed effects, and plot was used as random effect. The residuals were checked for normality assumptions. We conducted Tukey HSD post-hoc analysis to identify specific significant pairwise comparisons among month or vegetation overall.

#### Co-Occurrence Networks

We constructed co-occurrence networks to compare the community structures between the different vegetation types where repeated measures were addressed by aggregating data within plots to assess how microbial assemblies are affected by the grass-to-shrub transition. We used the SPIEC-EASI (SParse InversE Covariance Estimation for Ecological Association Inference) methodology [48]. To build and compare networks, we used the R packages microeco (version 1.1.0) and meconetcomp (version 0.3.0) [49, 50]. For both the bacterial and fungal datasets, the ASV table was filtered using a threshold of 0.0001 to reduce any ASVs with very low abundances. The datasets were then subset by vegetation type to build individual co-occurrence networks. Each dataset was further filtered with a threshold of 0.0007 which removed low abundance ASVs to improve downstream network interpretations. Additionally, we calculated Spearman’s Rank correlation coefficient to obtain correlations between the ASVs. These data were then used to calculate the network using the SPIEC-EASI’s Meinshausen-Buhlmann’s neighborhood selection option. The network modules were calculated using the “cluster_fast_greedy” parameter. The nodes, representing the ASVs, and edges of the networks, representing positive or negative relationships, were calculated and compared across vegetation types.

The networks were exported from R and imported into Gephi (version 0.10), a network construction software [51]. In Gephi, the nodes of the network were colored by module, the edges were colored by positive or negative ASV associations, the node size was determined by how frequently that ASV occurred in the data, and the edge thickness was determined by how frequently the ASV connection occurred. The network layout was constructed using ForceAtlas 2 [52].

#### Indicator Species Analyses

We conducted indicator species analysis using the R package indicspecies (version 1.7.14) [53]. The “multipatt” function was utilized with the function “IndVal.g.” For this, ASV tables were limited to the ASVs that had a frequency greater than 25% to assess more common indicators in the samples. The number of identified indicators were reduced using the “A” parameter (i.e., how unique the ASV is to a vegetation type) and the “B” parameter (i.e., the frequency of the ASV in that vegetation type). For this study, the identified indicators were limited to include species that were found almost only in that vegetation type (i.e., “A” for fungal indicators ≥ 90% and “A” for bacterial indicators ≥ 60%) and were in at least half of every sample of that vegetation type (i.e., “B” for fungal indicators ≥ 50% and “B” for bacterial indicators ≥ 60%). This provided a more restricted list of indicator species that were more informative about the community. Additionally, FunGUILD v.1.1 and FARPROTAX v.1.2.10 were utilized to identify the functional groupings of the fungal and bacterial indicators, respectively [54, 55].

#### Differential Abundance Analyses (DAA)

To analyze abundance differences of the taxonomic groups, we conducted differential abundance (DA) analysis using ANCOM-BC2 in the R package ANCOMBC (version 2.2.2) [56, 57] which can account for repeated measures. Using this package, pairwise comparisons between each vegetation type and each sampling month were conducted. This returns the natural log-fold changes between the two compared groups and indicates differentially abundant taxonomic classifications (i.e., order and class) between the groups. Order level was used for fungi and class level was used for bacteria as 102 fungal orders and 95 bacterial classes are represented in this dataset; bacterial orders were far too numerous (200 orders) for the purposes of this analysis. Additionally, only taxa that passed sensitivity analyses were used for data interpretation. Pseudo-counts are utilized in the ANCOM-BC2 algorithm to account for zeros in the data that would disrupt the logarithms. The sensitivity analysis tests to see if any significant results are false discoveries due to the pseudo-count [58].

All scripts, ASV tables, and statistical results are available at https://github.com/EEmbury/Embury_RomeroOlivares_2025 and via EDI (DOI: 10.6073/pasta/197e86c0503c9142edeaa871ffcc1ce7).

## Results

**Fig. 2:**
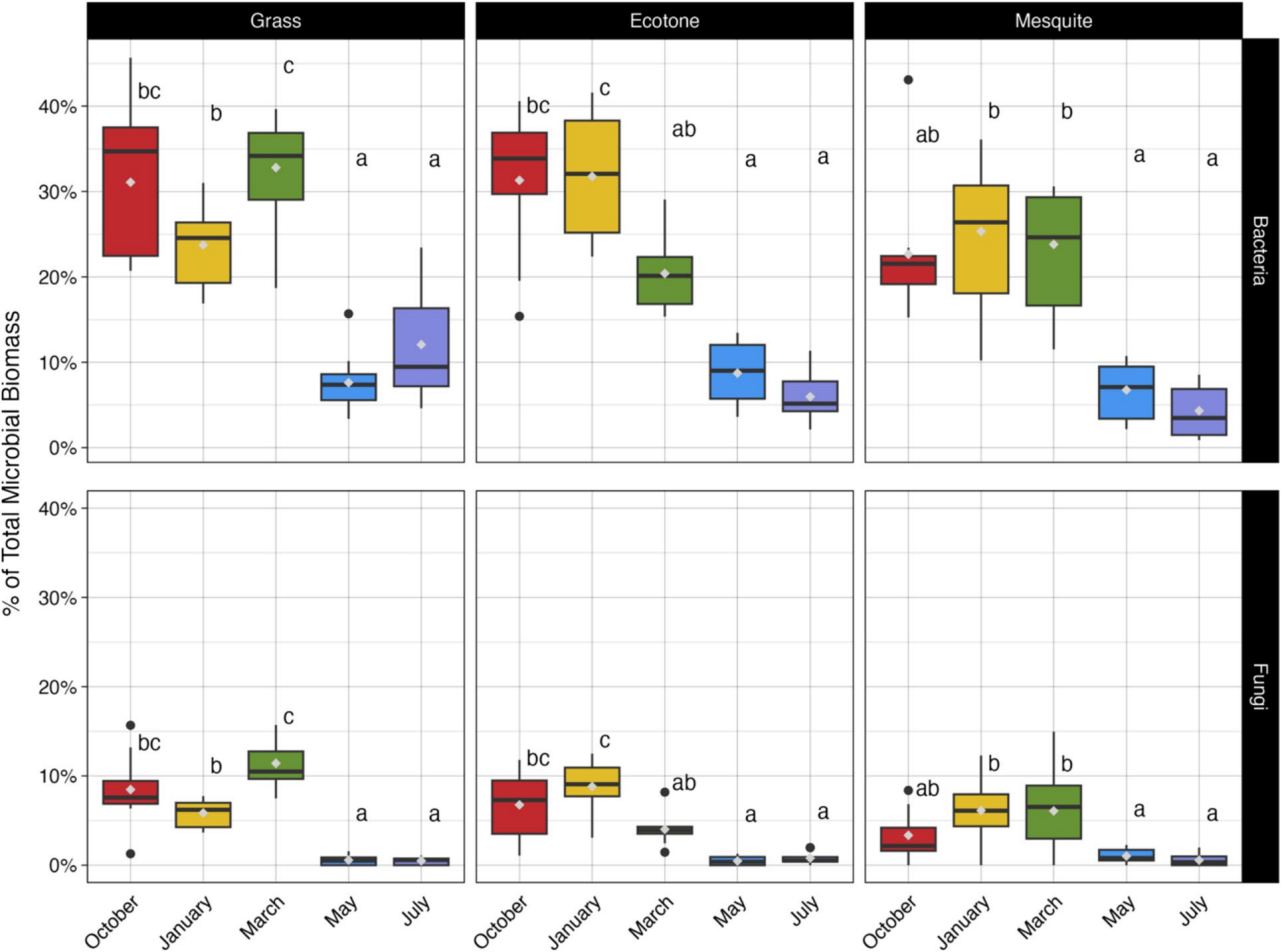
Bacterial and fungal percentage of total microbial biomass by vegetation type. In the box and whisker plots, the boxes show the upper and lower quartiles, and the whiskers show the highest and lowest data extremes. Outliers are indicated by the black points, the median is shown by the black bar within each box, and the mean is shown by the gray point within each box. Different letters show significant differences of each vegetation and microbial biomass comparison

To answer our first question, how does the microbial community respond in space and time and when considering biotic (i.e., vegetation) and abiotic (i.e., seasonality) factors? We found that the fungal and bacterial biomass differed significantly across sampling months and vegetation types (Fig. 2). We found significant differences in the microbial biomass by sampling month (fungi, *p* = 2, *p* = 2.00 × 10^−16^; bacteria, *p* = 2.10 × 10^−16^), vegetation type (fungi, *p* = 0.002; bacteria, *p* = 0.009), and their interaction (fungi, *p* = 7.15 × 10^−7^; bacteria, *p* = 0.0003) (Supplementary Table 1). Specifically, there was significantly less bacterial biomass between the ecotone (19.64%, *p* = 0.043) and the grass (21.46%, *p* = 0.0007) sites in comparison to mesquite sites (16.41%) (Supplementary Tables 2 and 3). There were no significant differences between the ecotone and the grass site. Moreover, there was significantly more fungal biomass in the grass site (5.36%, *p* = 0.001) compared to the mesquite site (3.43%). However, there were no significant differences in the fungal biomass between the grass and mesquite sites, when compared to the ecotone site (Supplementary Tables 2 and 4). In terms of monthly variation, we found that the bacterial and fungal biomass oscillated significantly across time (Supplementary Tables 5 and 6) and vegetation type (Supplementary Tables 3 and 4). In correlation assessments (Tables 1 and 2), temperature and relative humidity are significantly correlated with changes in both bacterial and fungal biomass. Additionally, C:N ratios are correlated with bacterial biomass changes.

**Table 1:** Pearson’s correlation of bacterial biomass and environmental variables. Significant values shown in bold

	***t***	df	95% CI lower	95% CI upper	Cor.coefficient	***p***-value
Temperature	− 11.052	133	− 0.7707629	− 0.5921811	− 0.6919104	** < 2.2*e* − 16**
Precipitation	0.66248	133	− 0.1126998	0.2241347	0.05734938	0.5088
Humidity	9.6713	133	0.5312902	0.7320485	0.6425671	** < 2.2*e* − 16**
pH	1.9482	133	− 0.0024588	0.32634055	0.1665675	0.0535
C:N	2.9509		0.08238332	0.40009027	0.24789	**0.003746**

**Table 2:** Pearson correlation of fungal biomass and environmental variables. Significant values shown in bold

	***t***	df	95% CI lower	95% CI upper	Cor.coefficient	***p***-value
Temperature	− 9.2904	133	− 0.7199857	− 0.5127322	− 0.6273428	**3.91*E* − 16**
Precipitation	− 0.96628	133	− 0.24894	0.08668531	− 0.0834947	0.3357
Humidity	6.0841	133	0.3231235	0.5891197	0.4666067	**1.17*E* − 08**
pH	1.7143	133	− 0.022484	0.30832928	0.1470317	0.08881
C:N	1.0084	133	− 0.0830719	0.252351	0.08710783	0.3151

**Fig. 3:**
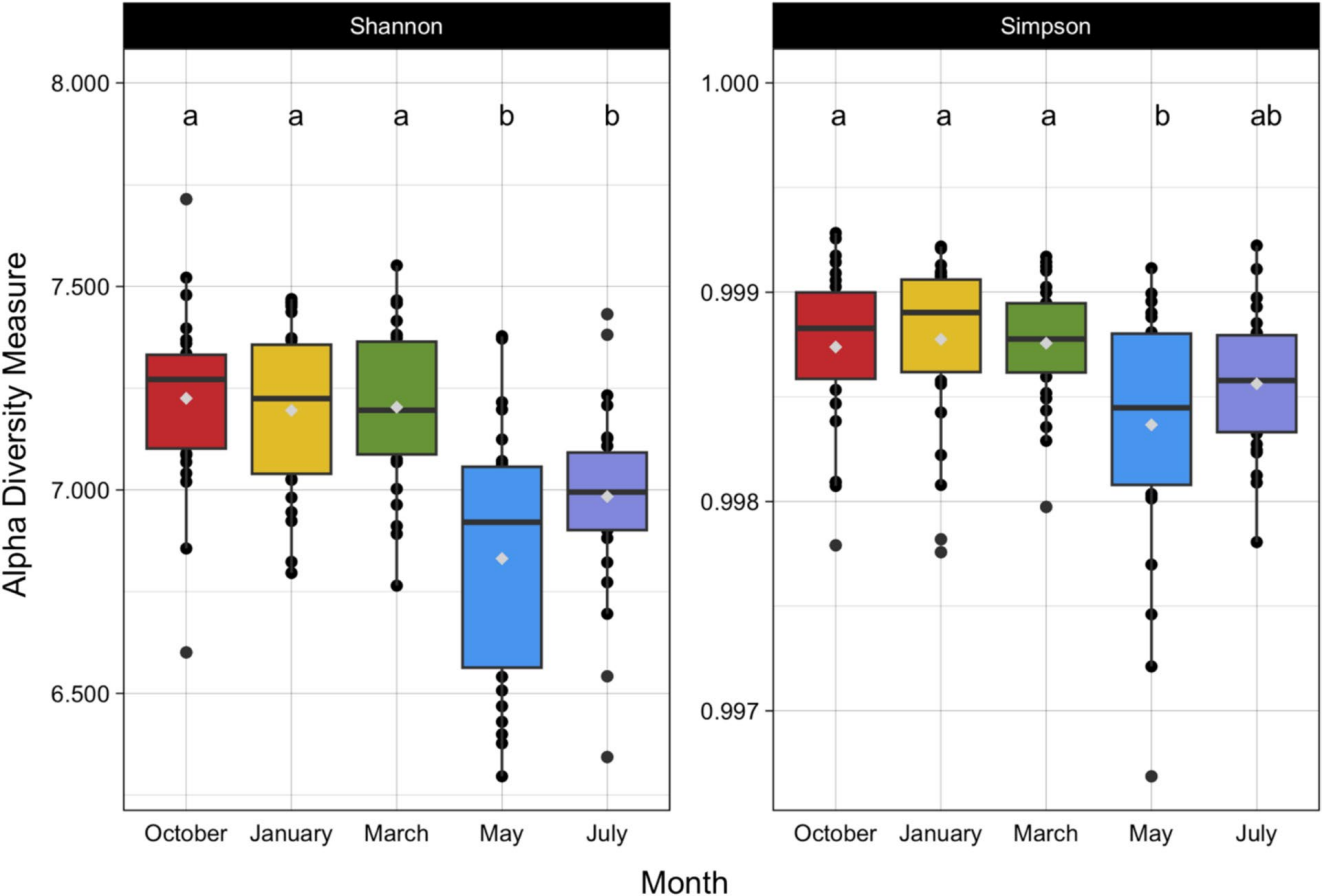
Shannon-Weiner and Simpson bacterial alpha diversity by sampling month. In the box and whisker plots, the boxes show the upper and lower quartiles, and the whiskers show the highest and lowest data extremes. Outliers are indicated by the black points separate from the whiskers, the median is shown by the black bar within each box, and the mean is shown by the gray point within each box. Different letters show significant differences between months for each diversity metric

Building upon the findings related to our first question, which asked how the microbial community responds in space and time considering biotic (i.e., vegetation) and abiotic (i.e., seasonality) factors. We found that bacterial diversity was impacted by seasonality, while fungal diversity was influenced by vegetation. Bacterial alpha diversity differed significantly by month whereas fungal alpha diversity differed significantly by vegetation type. Both the Shannon-Weiner and Simpson diversity metrics returned the same overall statistical results (Tables 3, 4, 5, and 6). The alpha diversity of the bacterial samples varied significantly by month in both the Shannon-Weiner (*p* = 9.08 × 10^−10^) and Simpson metrics (*p* = 0.001) (Tables 3 and 4). Yet, the Tukey HSD post-hoc analysis differed between the Shannon-Weiner and Simpson metrics. For the Shannon-Weiner diversity metric, the values in October, January, and March differed significantly from those in July and May (Fig. 3), whereas for the Simpson diversity metric, the values in October, January, and March only differed significantly from May (Fig. 3).

**Table 3:** Bacterial Shannon-Weiner diversity mixed effects model results. Significant values shown in bold

	df	Sum Sq	Mean Sq	***F***	***p***-value
Month	4	3.1845	0.79613	14.6667	**9.08 × 10**^**−10**^
Vegetation	2	0.0285	0.01426	0.2628	0.7694
Month × vegetation	8	0.646	0.08080	1.4885	0.1684

**Table 4:** Bacterial Simpson diversity mixed effects model results. Significant values shown in bold

	df	Sum Sq	Mean Sq	***F***	***p***-value
Month	4	3.23** × **10^−6^	8.09 × 10^−7^	4.786	**0.0012**
Vegetation	2	4.174** × **10^−7^	2.08 × 10^−7^	1.234	0.2948
Month × vegetation	8	1.13** × **10^−6^	1.42 × 10^−7^	0.83	0.5695

**Table 5:** Fungal Shannon-Weiner diversity mixed effects model results. Significant values shown in bold

	df	Sum Sq	Mean Sq	***F***	***p***-value
Month	4	0.153	0.0383	0.129	0.9584
Vegetation	2	1.84	0.9208	3.822	**0.0246**
Month × vegetation	8	2.67	0.3339	1.3863	0.2094

**Table 6:** Fungal Simpson diversity mixed effects model results. Significant values shown in bold

	df	Sum Sq	Mean Sq	***F***	***p***-value
Month	4	0.0019	0.00049	0.1281	0.9719
Vegetation	2	0.0323	0.016189	4.2042	**0.0172**
Month × vegetation	8	0.0391	0.004897	1.2716	0.2678

**Fig. 4:**
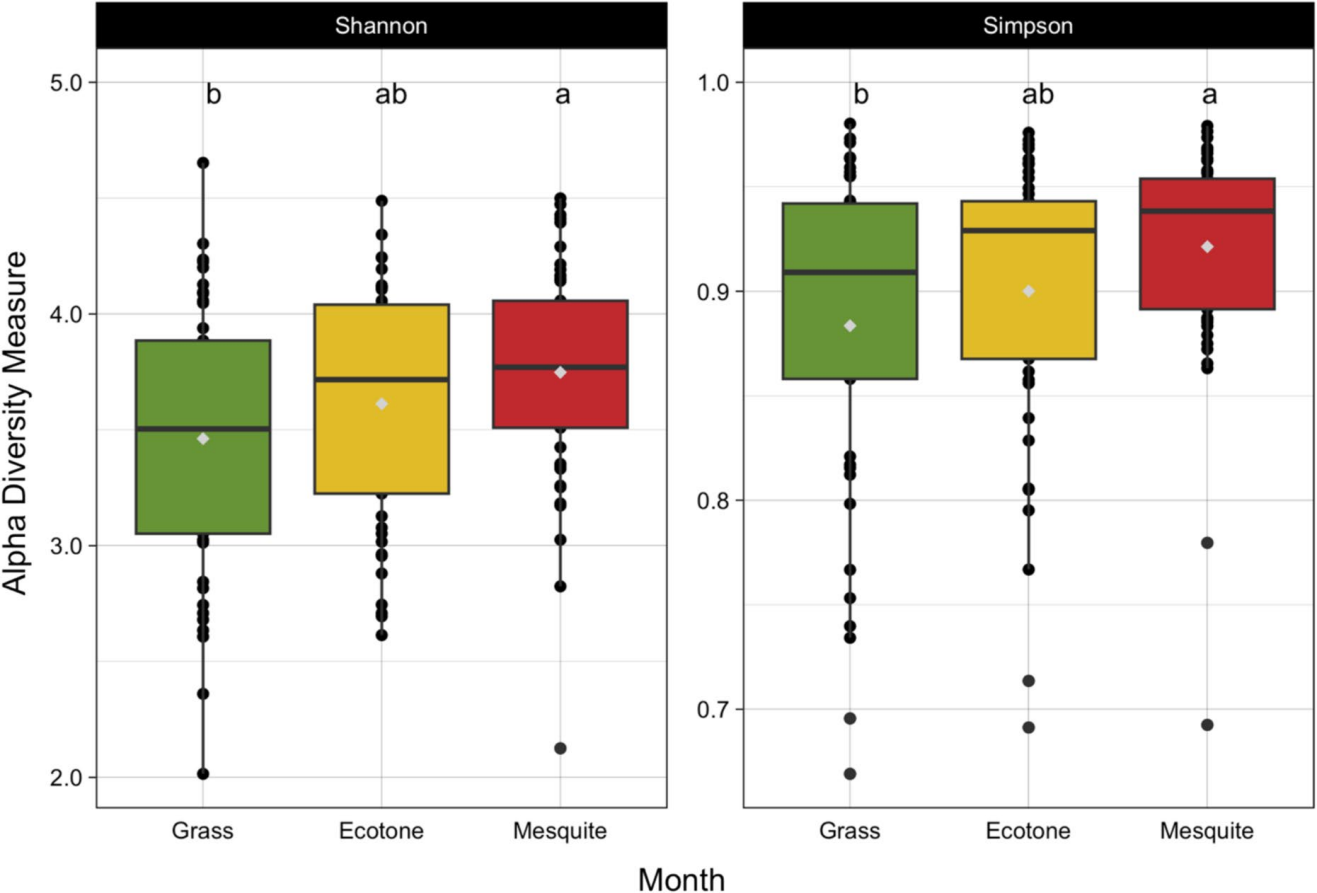
Shannon-Weiner and Simpson fungal alpha diversity by vegetation type. In the box and whisker plots, the boxes show the upper and lower quartiles, and the whiskers show the highest and lowest data extremes. Outliers are indicated by the black points separate from the whiskers, the median is shown by the black bar within each box, and the mean is shown by the gray point within each box. Different letters show significant differences between vegetation types for each diversity metric

The alpha diversity of the fungal samples varied significantly by vegetation type in both the Shannon-Weiner (*p* = 0.02) and Simpson (*p* = 0.01) diversity metrics (Tables 5 and 6). Both the Shannon-Weiner and Simpson diversity metrics showed that the mesquite site differed significantly from the grass site based on Tukey HSD post-hoc results (Fig. 4).

**Fig. 5:**
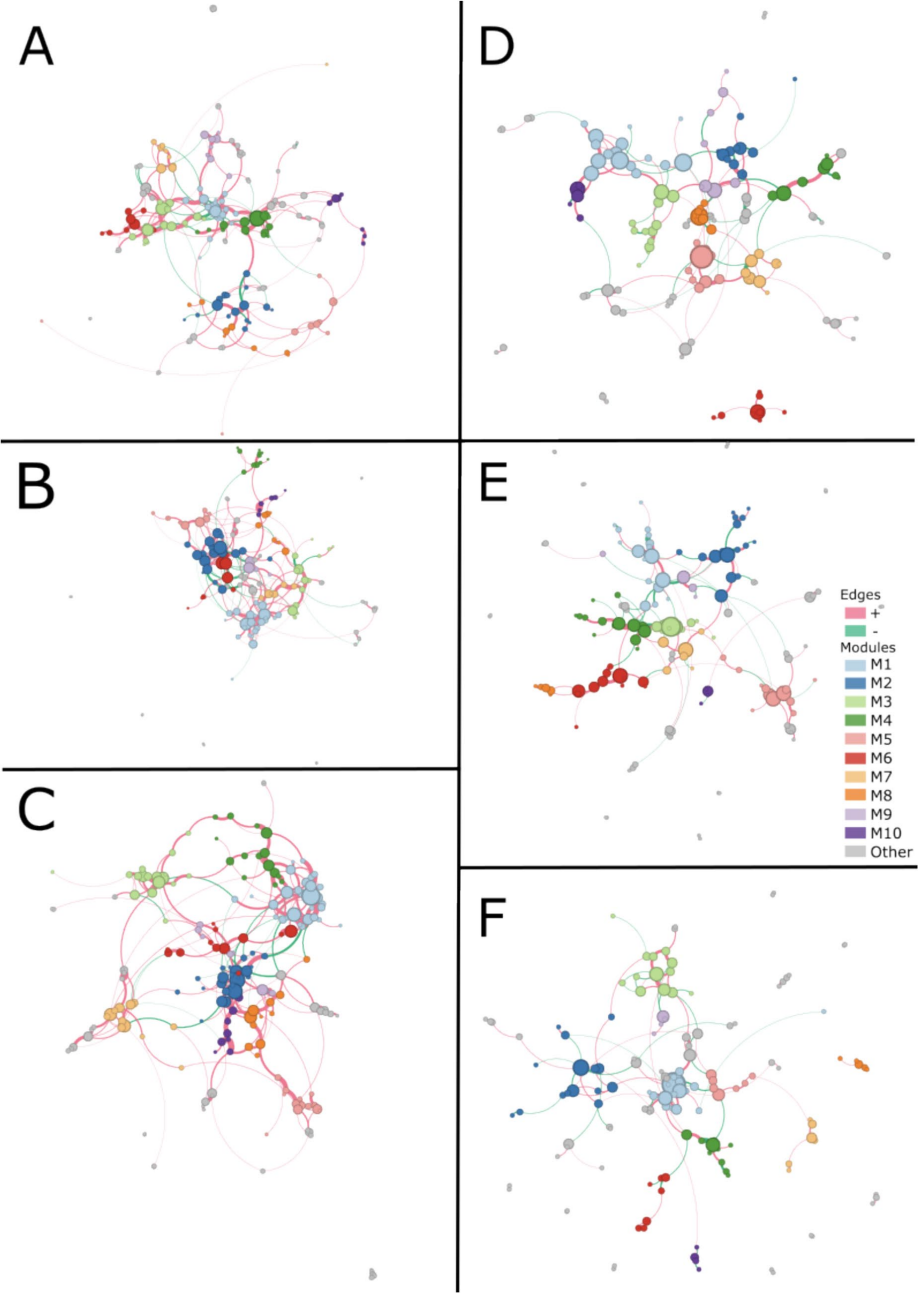
In co-occurrence networks, circles indicate nodes and connecting lines indicate edges. Circles of the same color are members of the same module and line color indicates a positive or negative relationship between nodes. The node size represents how frequently that ASV occurred in the data, and the edge thickness represents how frequently the ASV connection occurred. **A** Bacteria grass site network. The 10 highlighted modules account for 70.11% of the nodes. 88.3% positive edges, and 11.7% negative edges. **B** Bacteria ecotone site network. The 10 highlighted modules accounted for 74.85% of the nodes. 87.02% positive edges, and 12.98% negative edges. **C** Bacteria mesquite site network. The 10 highlighted modules accounted for 78.56% of the nodes. 94.3% positive edges, and 5.7% negative edges. **D** Fungi grass site network. The 10 highlighted modules accounted for 68.48% of the nodes. 72.73% positive edges, and 27.27% negative edges. **E** Fungi ecotone site network. The 10 highlighted modules accounted for 69.87% of the nodes, 71.58% positive edges, and 28.42% negative edges. **F** Fungi mesquite site network. The 10 highlighted modules accounted for 64.49% of the nodes. 68.79% positive edges, and 31.21% negative edges

To answer our second question, how do microbial networks assemble in a grassland-to-shrubland gradient? We found that fungal communities assemble more unique networks in each vegetation type when compared to bacterial networks which were more uniform across the vegetation types (Fig. 5). Networks showed that the fungal community is more unique by vegetation compared to bacteria, as few fungal nodes were shared across all vegetation types in comparison to bacteria which shared more. However, interactions were highly variable in both fungi and bacteria in response to vegetation type. Specifically, 39.4% of the bacterial nodes were shared across all three vegetation types, 11.4% were unique to the grass site, 6.1% were unique to the ecotone site, and 23.1% were unique to the mesquite site. Contrastingly, 11.4% of the fungal nodes were shared across all three vegetation types, 24.7% were unique to the grass site, 22.4% were unique to the ecotone site, and 21.4% were unique to the mesquite site (Table 7). Overall, based on Bray–Curtis dissimilarity metrics, on average, 30% of the network nodes and 92% of the edges differed among the vegetation types in bacteria, while in fungi, on average, 62% of the network nodes and 98% of the edges differed between vegetation types (Supplementary Tables 7, 8, 9, and 10).

**Table 7:** Percentage of nodes and edges connected to different vegetation types on the bacterial and fungal co-occurrence networks

	Bacterial network		Fungal network	
	Percentage of nodes	Percentage of edges	Percentage of nodes	Percentage of edges
Only in grass	11.40% (*30*)	29.30% (*230*)	24.70% (*76*)	33% (*166*)
Only in mesquite	23.10% (*61*)	36.30% (*285*)	21.40% (*66*)	30.20% (*152*)
Only in ecotone	6.10% (*16*)	28.10% (*221*)	22.40% (*69*)	34.20% (*172*)
Shared between grass & mesquite	6.80% (*18*)	1.10% (*9*)	3.20% (*10*)	0.40% (*2*)
Shared between grass & ecotone	8.30% (*22*)	2.40% (*19*)	8.10% (*25*)	1.60% (*8*)
Shared between mesquite & ecotone	4.90% (*13*)	1.90% (*15*)	8.80% (*27*)	0.60% (*3*)
Shared in all vegetation types	39.40% (*104*)	0.90% (*7*)	11.40% (*35*)	0% (*0*)

**Fig. 6:**
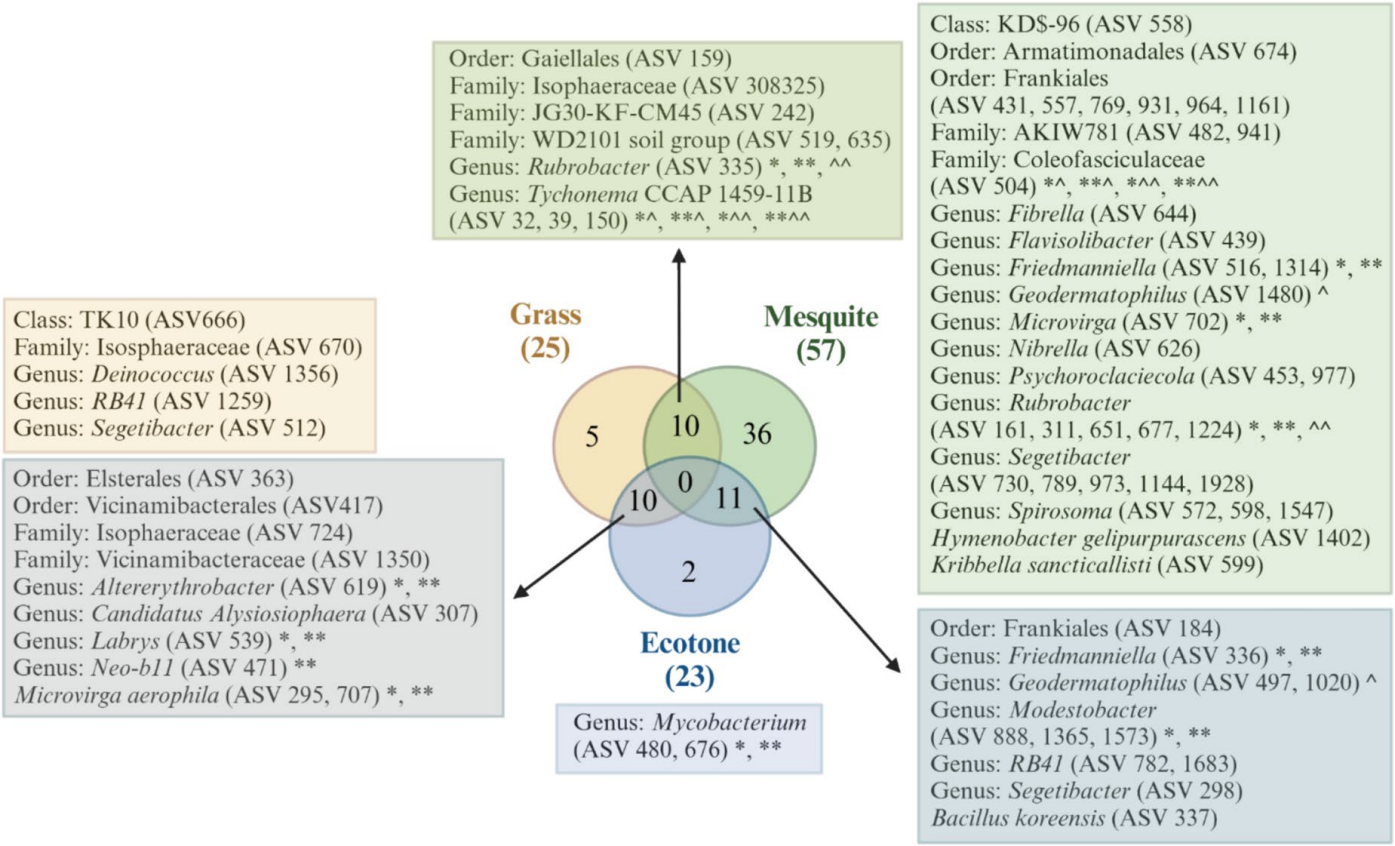
Venn diagram shows bacterial indicator species of each site and overlap between sites. Boxes show indicator species to the highest known taxonomic level. Symbols represent functional grouping as identified by FARPROTAX, *aerobic chemoheterotrophy, **chemoheterotrophy, ^manganese oxidation, ^^nitrate reduction, *^oxygenic photoautotrophy, **^photoautotrophy, *^^phototrophy, and **^^photosynthetic cyanobacteria. Venn diagram developed with interactivenn.net and illustrated using Biorender (license number CR27WNN70C)

**Fig. 7:**
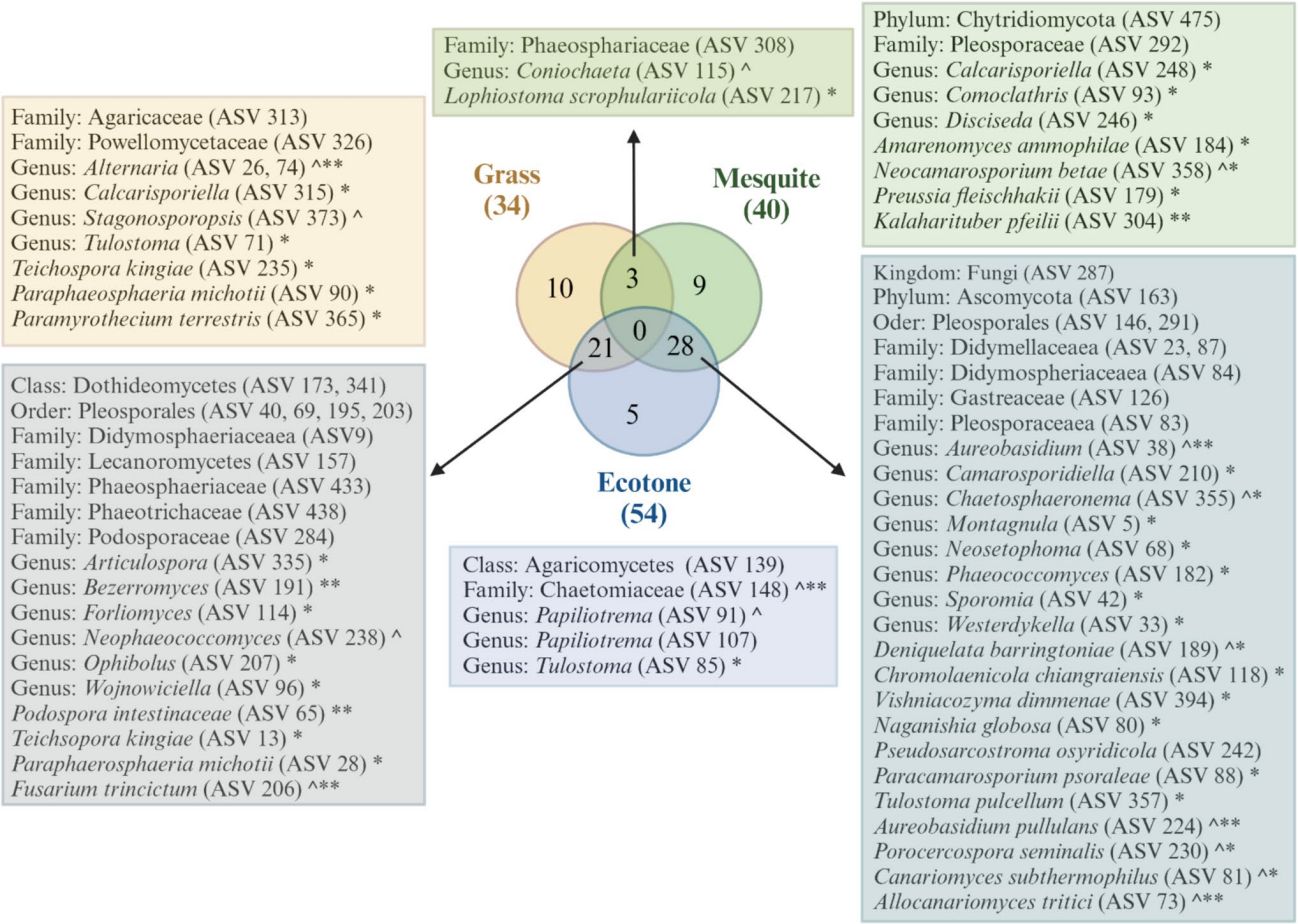
Venn diagram shows fungal indicator species of each site and overlap between sites. Boxes show indicator species to the highest known taxonomic level. Symbols represent trophic modes as identified by FunGUILD, *saprotroph, **saprotroph-symbiotroph, ^pathotroph, ^*pathotroph-saprotroph, and ^**pathotroph-saprotroph-symbiotroph. Venn diagram developed with interactivenn.net and illustrated using Biorender (license number TV27WNDB2S)

To answer our third question, which microbial taxa may be responding strongly to shrub encroachment and/or playing a crucial role in facilitating this phenomenon in the northern extent of the Chihuahuan Desert? We found high levels of overlap between indicators of vegetation types, meaning that there were minimal taxa that were found to be key indicators of the shrub encroachment phenomenon. In the indicator species analysis, few indicator species were unique to a site. That is, most indicator species were shared between grass and ecotone, or mesquite and ecotone, for both bacteria and fungi. Briefly, there were 74 bacterial indicator species, of which 28 were matched with a functional grouping with FARPROTAX [54]. The majority of them (35 taxa) were indicator species of mesquite sites (Fig. 6). Additionally, there were 76 fungal indicator species, of which 59 were matched with a functional grouping with FunGUILD [55]. The majority of indicators were indicators of two vegetation types, 28 taxa were indicators of the mesquite and ecotone sites, and 24 were taxa of grass and ecotone sites (Fig. 7).

To further answer our third question, which microbial taxa may be responding strongly to shrub encroachment and/or playing a crucial role in facilitating this phenomenon in the northern extent of the Chihuahuan Desert? We found numerous bacterial classes and fungal orders that do respond strongly to differences in the dominant vegetation. The DAA revealed that the degree of change in the microbial community is higher in fungi across sites compared to the bacteria. That is, the fold change of fungal orders was more pronounced than for bacterial classes across all sites.

**Fig. 8:**
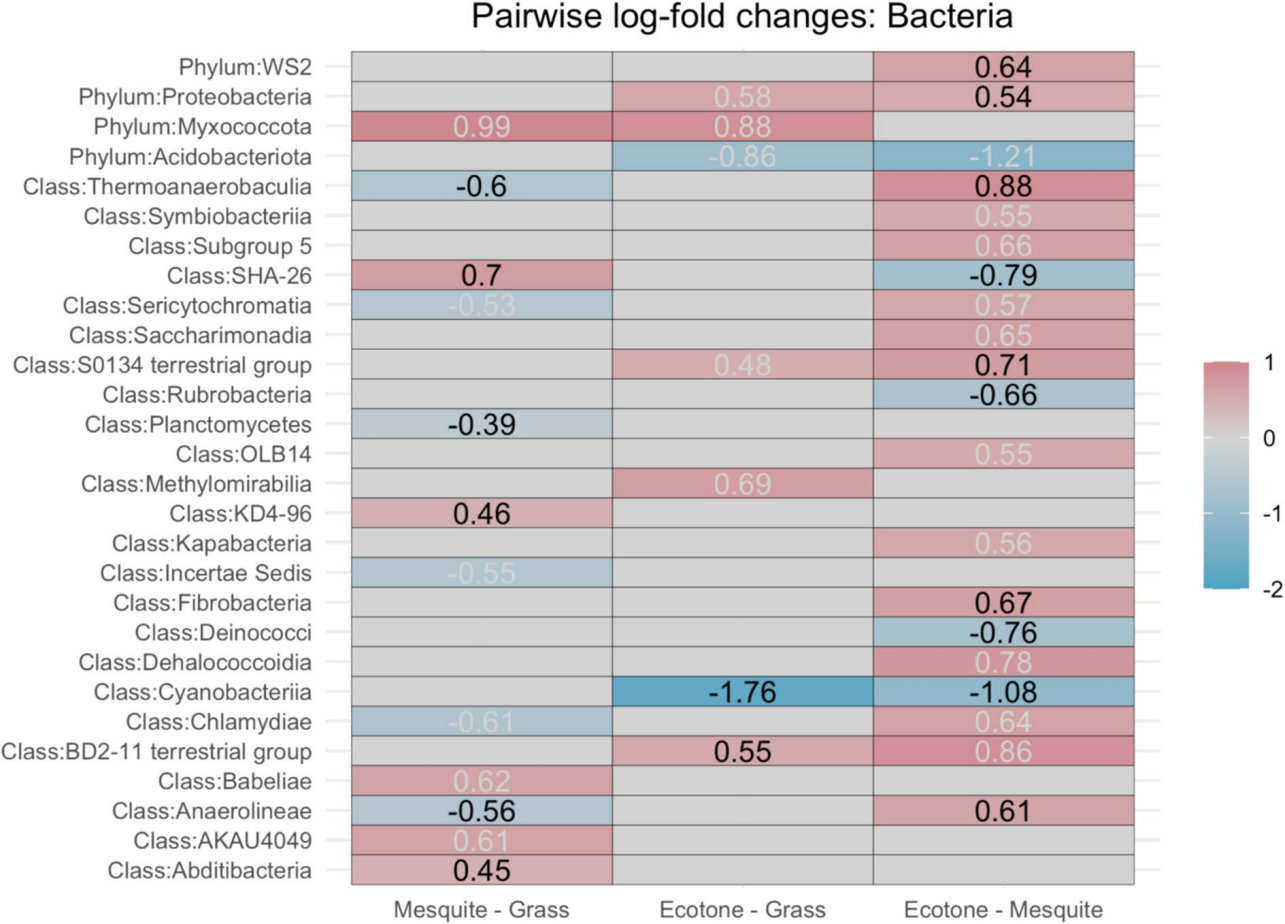
Differentially abundant bacterial classes. Red indicates a positive natural log-fold change in abundance; blue indicates a negative natural log-fold change in abundance. The first column compares the mesquite site to the grass site. The second column compares the ecotone site to the grass site. The third column compares the ecotone site to the mesquite site. Black text indicates the taxa passed the pseudo-count sensitivity analysis; grey text indicates it did not pass the pseudo-count sensitivity analysis

Bacteria had 16 classes with significant natural log-fold changes between vegetation types that passed sensitivity analyses, some of which overlapped between comparisons (e.g., Cyanobacteria had a negative fold change in both the ecotone-grass and ecotone-mesquite comparisons) (Fig. 8). Six classes of the 16 were differentially abundant in the mesquite compared to the grass site, three of which were negative changes (Thermoanaerobaculia, Planctomycetes, and Anaerolineae), and three were positive (SHA-26, KD4-96, and Abditibacteria). Two classes of the 15 were differentially abundant in the ecotone compared to the grass site, one was a negative change (Cyanobacteriia), and one was a positive change (BD2-11 terrestrial group). Eight classes of the 16 were differentially abundant in the ecotone compared to the mesquite site, and four were positive changes (Thermoanaerobaculia, S0134 terrestrial group, Fibrobacteria, and Anaerolineae), and four were negative changes (SHA-26, Rubrobacteria, Deinococci, and Cyanobacteriia).

**Fig. 9:**
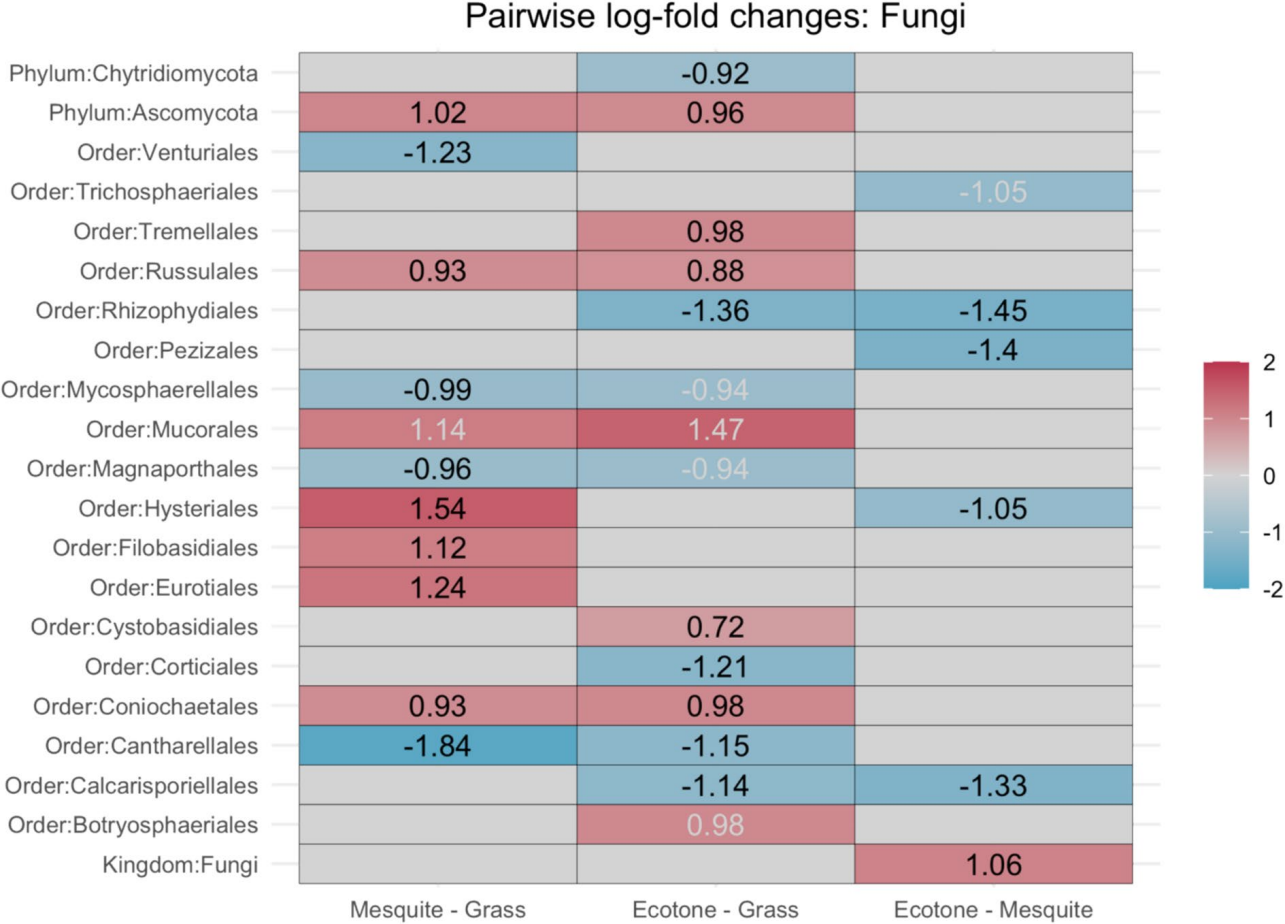
Differentially abundant fungal orders. Red indicates a positive natural log-fold change in abundance, blue indicates a negative natural log-fold change in abundance. The first column compares the mesquite site to the grass site. The second column compares the ecotone site to the grass site. The third column compares the ecotone site to the mesquite site. Black text indicates the taxa passed the pseudo-count sensitivity analysis; grey text indicates it did not pass the pseudo-count sensitivity analysis

For fungi, the DAA showed that 21 orders had significant natural log-fold changes between vegetation types that passed sensitivity analyses, some of which also overlapped (Fig. 9). Nine of the 21 orders were differentially abundant in the mesquite compared to the grass site, five were positive changes (Russulales, Hysteriales, Filobasidiales, Eurotiales, and Coniochaetales), and four were negative changes (Venturiales, Mycospherellales, Magnaporthales, and Cantharellales). Eight of the 21 orders were differentially abundant in the ecotone compared to the grass site, four of which were positive changes (Tremellales, Russulales, Cystobasidiales, and Coniochaetales), and four were negative changes (Rhyzophydiales, Corticiales, Cantharellales, and Calcarisopriellales). Four of the 15 orders (Rhizophydiales, Pezizales, Hysteriales, and Calcarisopriellales) were differentially abundant in the ecotone compared to the mesquite site and were all negative changes.

In the DA analysis of different sampling months, many bacterial classes and fungal orders were differentially abundant (Supplementary Tables 11 and 12) but a common trend across these differentially abundant taxa is that most of the differences occurred when a warm sampling month (May or July) was compared to a non-warm sampling month (October, January, or March). Only one non-warm sampling month comparison resulted in differentially abundant taxa for bacteria and one non-warm sampling month comparison, resulted in differentially abundant taxa for fungi (highlighted in Supplementary Tables 11 and 12). Notably, all non-warm month comparisons that led to the identification of differentially abundant taxa were between October and March.

## Discussion

We found support for our hypothesis that the microbial community would respond differently in space and time. Specifically, we found that the microbial communities in a grassland-to-shrubland gradient in our sites at the Jornada LTER in the Chihuahuan Desert are highly dynamic in space and time. Microbial biomass measurements show evidence of both temporal and spatial variations within the microbial community. Moreover, we hypothesized that bacteria would be more responsive to changes in abiotic factors while fungi would be more responsive to changes in biotic factors and that these responses would be reflected in the diversity of the community, and its microbial networks and abundance dynamics. Indeed, our work showed that grass sites had the highest percentage of both fungal and bacterial biomass (Supplementary Table 2), and all sites experienced a significant decrease in biomass in the summer months May and July (Fig. 2). In fact, there were over 10 times more fungal biomass and 3.5 times more bacterial biomass in the October/January/March sampling months when compared to May/July (Fig. 2 and Supplementary Table 13).

The decrease in biomass in the summer months can be tied to both abiotic and biotic controls on microbial communities in dryland systems. Several abiotic factors could potentially act as drivers of the microbial biomass such as temperature, humidity, precipitation, soil pH, and carbon-to-nitrogen ratios. We found that temperature and humidity are significant drivers of changes in microbial biomass (Tables 1 and 2). This is partially similar to what others have observed—sampling dates with the lowest soil moisture content as a result of low precipitation resulted in the lowest microbial biomass [22]. In our data, however, October and May both had above average levels of rainfall with 60.37 mm and 30.05 mm, respectively (Supplementary Table 14), but microbial biomass trends differed. If rainfall alone was the primary determining factor in microbial biomass levels, we would expect to see similar biomass trends in these two above-average precipitation months. Instead, we see variations in microbial biomass, demonstrating that rainfall alone is not the predominant driver of biomass alterations. Our conclusions are supported by Pearson’s correlation metrics, which did not identify a significant correlation between precipitation and biomass. Although a correlation between increases in precipitation and increases in microbial biomass may be true in forest ecosystems [e.g., 59], there is limited evidence that this holds true for dryland ecosystems [e.g., 60]. But even in forested ecosystems, precipitation has been shown to be highly influential on fungal and bacterial biomass patterns but on a shorter timescale of hours [61]. Finer scale sampling of days/hours would be required in our study system, to elucidate potential trends in biomass related directly to precipitation. Nonetheless, we show that seasonal variance of temperature and relative humidity is an important driver of changes in microbial biomass. Although water availability in drylands is typically thought of as a critical limiting factor for microbial communities, we show that relative humidity also plays an important role [62, 63]. Both fungal and bacterial biomass were significantly correlated with relative humidity. Relative humidity is often correlated with temperature, which we found to also play an important role in microbial biomass dynamics. Indeed, abiotic factors such as temperature, humidity, carbon and nitrogen ratios, and pH have been shown to be closely related to microbial community trends [21, 64–66].

Biotic influences such as dominant vegetation types also appear to influence the microbial biomass to different extents. We found higher percentage of both bacterial and fungal biomass in grass sites, while mesquite sites contained the lowest level of microbial biomass (Supplementary Table 2). The influence of the dominant plant on microbial biomass may be linked partially to the resource island phenomenon observed in drylands. Under-shrub soils have been shown to have higher levels of microbial biomass in comparison to interspace soils [22]. The mesquite site in this study was heavily dominated by interspace soils potentially explaining the lower microbial biomass. Additionally, as was noted previously, temperature and humidity were strongly correlated with microbial biomass in this study. Grass-dominated sites have been recorded having higher moisture content in summer months when compared to shrub-dominated locations as grass provides higher soil shading and limits evaporation leading to higher soil moisture [67]. The moisture changes provided by the grass could potentially explain why the grass sites had higher biomass when compared to the shrub dominated sites. Another notable potential biotic effect is the allelopathic properties of mesquite. Honey mesquite is known for having allelopathic properties that reduce the germination success of seedlings in laboratory settings [68]. In addition, honey mesquite’s relative, *P. juliflora*, is an aggressive invader due to its widely documented allelopathic properties that reduce seed germination of surrounding plants [69–71]. *Prosopis juliflora* allelopathy can inhibit bacterial growth, and there are many other documented cases where microbes are affected by plant allelopathy [72, 73]. Therefore, it is possible that mesquite allelopathy is impacting microbial communities and a contributing factor in the lower quantities of microbial biomass [74].

While fungal biomass measurements were highly impacted by seasons, fungal community assembly and diversity were not; instead, these were highly impacted by vegetation (Tables 5 and 6). Contrastingly, bacterial community assembly and diversity were more heavily influenced by seasons (Tables 3 and 4). This is in line with what others have found where abiotic and biotic pressures lead to differing responses in bacterial versus fungal communities [75–77]. This is not unexpected as fungal communities are closely tied to plants [78] and have undergone co-evolution to develop the mutualistic relationships that we observe today [79]. For example, mutualistic fungi receive carbon from symbiotic relationships with plants, and in drylands, mycorrhizal fungi can potentially act as a conduit for nutrient movement, forming a network between shrubs and biological soil crusts [80–82]. These relationships demonstrate how closely connected plants and fungi are, and although the emphasis of our study was not on mycorrhizal fungi, our results highlight how even non-symbiotic fungi can be influenced by plants, and vice versa.

Network and differential abundance analyses, as well as diversity indices, all support the findings that fungi are more impacted by vegetation type than bacteria. Network analyses showed that the different vegetation types foster unique fungal community assemblies and while unique bacterial communities also form, the trend is much smaller compared to fungal communities (Table 7). Previous research in the Jornada LTER showed that fungi are relatively resistant to abiotic disturbances, as a 70% reduction in incoming precipitation coupled with physical disturbance did not alter the fungal community composition but showed high spatial heterogeneity based on the presence of absence of vegetation [83]. This close relationship to plants was also evident in dissimilarity analyses of the microbial networks as there was higher dissimilarity between fungal community nodes than bacterial community nodes (Supplementary Tables 7 and 9).

The pairwise differential abundances analysis showed that more fungal orders had a more pronounced fold-change compared to most bacterial classes. So not only were there more differentially abundant fungal taxa, but also, the differences of the abundance between vegetation types were of a higher magnitude when compared to the changes observed in bacterial classes. Some of the fungal taxa with the strongest fold-change showed interesting patterns. For example, the abundance of Cantharellales was significantly lower at the mesquite and ecotone sites than at the grass site, and while it is difficult to make assumptions about the taxa at the order level, many members of the Cantharellales lack the ability to decompose lignin which would be found in the mesquite wood [84]. When looking at monthly variability, there were 15 bacterial classes that were differentially abundant and passed sensitivity analyses; five of the comparisons had a large degree of change. Contrastingly, only five fungal orders were differentially abundant and passed sensitivity analyses, yet all of these had also a large degree of change. Interestingly, while fungi had much fewer differentially abundant taxa across monthly comparisons, the magnitude of change was strong. When a change occurs in the fungal communities, it seems to be a substantial change whereas changes in bacterial communities seem to be of a smaller, yet still significant magnitude. Numerous studies have also recorded seasonal variability in bacterial communities across numerous ecosystems [85, 86] and drylands included [87, 88]. This variability can be linked to fluctuations in soil nutrient availability, soil moisture, and soil temperature [89, 90]. Indeed, we can see evidence of these biogeochemical influences in our data as the C:N ratio was significantly correlated with bacterial biomass but not fungal biomass.

Very few fungal indicator species (i.e., three) are found in both grass and mesquite sites in our sites at the Jornada LTER, highlighting once again the uniqueness of the fungal community by vegetation. In contrast, ten bacterial indicator species were shared between grass and mesquite sites. Although we did not observe any significant trends in the function of indicator species, some emerging patterns suggest that mesquite sites host microbes that may support plant growth, while grass sites harbor microbes that could either benefit or harm growth. For example, *Alternaria* [91] (Fig. 7) is an indicator species in the grass site and known to be both endophytic and pathogenic; when behaving as endophyte, it can increase stress tolerance in plants [92], but when behaving as pathogen it be devastating [93]. Given global change and its impact on microbial function, further gene expression analysis within the microbial community could offer valuable insights into the activity of the microbial community and whether they express beneficial or pathogenicity-related genes (e.g., [94]). In contrast, indicator species in the mesquite site ranged from stress tolerant microbes such as *Neocamarosporium* which is halotolerant [95], similarly to mesquite [96] to mycorrhizal such as *Kalaharituber* [97].

In our study, we found that microbial communities in a changing landscape transitioning from a grassland-dominated to a shrubland-dominated ecosystem in the Jornada LTER are well differentiated and that fungi seem to be strongly associated with plants, while bacteria seem to be more responsive to changes in the environment. Overall, our research at the Jornada LTER found support for our hypothesis that microbial communities would respond differently in space and time and that these responses are reflected in the diversity of the community and its microbial networks and abundance dynamics. Bacterial community diversity responded strongly to temporal changes (i.e., seasons), whereas fungal communities showed greater sensitivity to spatial differences (i.e., vegetation cover). These changes were documented through diversity metrics, variations in the abundance of fungal orders and bacterial classes across vegetation types, and organization of microbial networks. Through these results we can see that microbial communities are indeed influenced by shrub-encroachment, demonstrating the importance of increasing the representation of microbial communities into our studies of dryland systems. Our research is pivotal for understanding the future of dryland ecosystems, especially those undergoing shrub encroachment. By uncovering microbial mechanisms that are potentially driving this shift, we provide essential insights that reveal complex forces at play between the microbial community and biotic and abiotic factors. Our findings will be critical for guiding restoration efforts, particularly those looking to incorporate microbial-mediated solutions.

## Supplementary Information

Below is the link to the electronic supplementary material.

Supplementary Material 1 (DOCX 533 KB)

## Data Availability

Data will be permanently archived via Environmental Data Initiative and NCBI. All collected data, scripts, ASV tables, and statistical results are available at https://github.com/EEmbury/Embury_RomeroOlivares_2025 and via EDI (DOI: https://doi.org/10.6073/pasta/197e86c0503c9142edeaa871ffcc1ce7). DNA demultiplexed sequences and metadata were deposited in NCBI (accession numbers: PRJNA1242850 and PRJNA1242890).
