# Supplementary material for: Fungi Follow Flora, Bacteria Track the Seasons: A Tale of a Changing Landscape
Source: Microb Ecol. 2025 Jun 20;88:68. doi: 10.1007/s00248-025-02568-3 (PMC12178982; doi:10.1007/s00248-025-02568-3)
Supplement: Supplementary file 1 [file 248_2025_2568_MOESM1_ESM.docx]

**SUPPLEMENTARY MATERIAL**

Supplementary Figure 1. Temperature and relative humidity data collected with iButtons at the Jornada LTER during 2021-2022 and used as preliminary data to determine environmental trends and selection of sampling dates for the current study. Pink boxes indicate the months selected for sampling.

Supplementary Figure 2. Average cover of grass, mesquite, and bare soil in study sites.

Supplementary Table 1. Results of the mixed effects model for the bacterial and fungal biomass percentages. Significant values shown in bold.

|  |  | df | Sum Sq | Mean Sq | F | p-value |
| --- | --- | --- | --- | --- | --- | --- |
| Bacterial Biomass | Vegetation | 2 | 586.3 | 293.17 | 7.432 | **0.0009** |
|  | Month | 4 | 12220 | 3055.16 | 77.4584 | **2.0x10^-16^** |
|  | Month x Vegetation | 8 | 1263 | 157.95 | 4.0045 | **0.0003** |
| Fungal Biomass | Vegetation | 2 | 85.44 | 42.718 | 6.2066 | **0.002** |
|  | Month | 4 | 1222.06 | 305.514 | 44.3896 | **2.0x10^-16^** |
|  | Month x Vegetation | 8 | 349.35 | 43.669 | 6.3448 | **7.54x10^-7^** |

Supplementary Table 2. Average PLFA percentage in different vegetation types.

|  | Bacterial PLFA (%) | Fungal PLFA (%) |
| --- | --- | --- |
| Grass | 21.46 | 5.36 |
| Ecotone | 19.64 | 4.25 |
| Mesquite | 16.41 | 3.43 |

Supplementary Table 3: Tukey HSD post-hoc analysis of bacterial biomass by vegetation. Significant values shown in bold.

|  | estimate | SE | df | t ratio | p-value |
| --- | --- | --- | --- | --- | --- |
| Grass x Ecotone | 1.82 | 1.33 | 118 | 1.377 | 0.3564 |
| Grass x Mesquite | 5.04 | 1.32 | 118 | 3.807 | **0.0007** |
| Ecotone x Mesquite | -3.22 | 1.32 | 118 | -2.431 | **0.0434** |

Supplementary Table 4: Tukey HSD post-hoc analysis of fungal biomass by vegetation. Significant values shown in bold.

|  | estimate | SE | df | t ratio | p-value |
| --- | --- | --- | --- | --- | --- |
| Grass x Ecotone | 1.933 | 0.553 | 118 | 3.495 | 0.0019 |
| Grass x Mesquite | 1.933 | 0.553 | 118 | 3.495 | **0.0019** |
| Ecotone x Mesquite | -0.752 | 0.553 | 118 | -1.360 | 0.3650 |

Supplementary Table 5: Tukey HSD post-hoc analysis of bacterial biomass by month. Significant values shown in bold.

|  | estimate | SE | df | t ratio | p-value |
| --- | --- | --- | --- | --- | --- |
| January-July | 19.492 | 1.71 | 118 | 11.403 | **<0.0001** |
| January-March | 1.525 | 1.71 | 118 | 0.892 | 0.8992 |
| January-May | 19.245 | 1.71 | 118 | 11.259 | **<0.0001** |
| January-October | -1.404 | 1.71 | 118 | -0.821 | 0.9235 |
| July-March | -17.967 | 1.71 | 118 | -10.511 | **<0.0001** |
| July-May | -0.247 | 1.71 | 118 | -0.144 | 0.9999 |
| July-October | -20.89 | 1.71 | 118 | -12.225 | **<0.0001** |
| March-May | 17.72 | 1.71 | 118 | 10.367 | **<0.0001** |
| March-October | -2.094 | 1.71 | 118 | -1.714 | 0.4296 |
| May-October | -20.649 | 1.71 | 118 | -12.081 | **<0.0001** |

Supplementary Table 6: Tukey HSD post-hoc analysis of fungal biomass by month. Significant values shown in bold.

|  | estimate | SE | df | t ratio | p-value |
| --- | --- | --- | --- | --- | --- |
| January-July | 6.317 | 0.714 | 118 | 8.847 | **<0.0001** |
| January-March | -0.2378 | 0.714 | 118 | -0.333 | 0.9973 |
| January-May | 6.2293 | 0.714 | 118 | 8.724 | **<0.0001** |
| January-October | 0.7396 | 0.714 | 118 | 1.036 | 0.8382 |
| July-March | -6.5548 | 0.714 | 118 | -9.180 | **<0.0001** |
| July-May | -0.0878 | 0.714 | 118 | -0.123 | 0.9999 |
| July-October | -5.5774 | 0.714 | 118 | -7.811 | **<0.0001** |
| March-May | 6.4670 | 0.714 | 118 | 9.057 | **<0.0001** |
| March-October | 0.9774 | 0.714 | 118 | 1.369 | 0.6487 |
| May-October | -5.4896 | 0.714 | 118 | -7.688 | **<0.0001** |

Supplementary Table 7. Bray-Curtis Dissimilarity distances that represent overall differences in nodes between the bacterial networks.

|  | Grass | Ecotone | Mesquite |
| --- | --- | --- | --- |
| Grass | 0 | 0.2340426 | 0.3405405 |
| Ecotone | 0.2340426 | 0 | 0.3333333 |
| Mesquite | 0.3405405 | 0.3333333 | 0 |

Supplementary Table 8. Bray-Curtis Dissimilarity distances that represent overall differences in edges between the bacterial networks.

|  | Grass | Ecotone | Mesquite |
| --- | --- | --- | --- |
| Grass | 0 | 0.9013283 | 0.9449225 |
| Ecotone | 0.9013283 | 0 | 0.9238754 |
| Mesquite | 0.9449225 | 0.9238754 | 0 |

Supplementary Table 9. Bray-Curtis Dissimilarity of overall differences in nodes between the fungal networks.

|  | Grass | Ecotone | Mesquite |
| --- | --- | --- | --- |
| Grass | 0 | 0.602649 | 0.6830986 |
| Ecotone | 0.602649 | 0 | 0.5782313 |
| Mesquite | 0.6830986 | 0.5782313 | 0 |

Supplementary Table 10. Bray-Curtis Dissimilarity of overall differences in edges between the fungal networks.

|  | Grass | Ecotone | Mesquite |
| --- | --- | --- | --- |
| Grass | 0 | 0.9554318 | 0.987988 |
| Ecotone | 0.9554318 | 0 | 0.9823529 |
| Mesquite | 0.987988 | 0.9823529 | 0 |

Supplementary Table 11. Bacterial classes that are differentially abundant across months and passed sensitivity analyses. “lfc” is the natural log-fold change, “se” is the standard error, and “W” is the test statistic.

| Class | lfc | se | W | p value | p adj. | Month |
| --- | --- | --- | --- | --- | --- | --- |
| Abditibacteria | -0.55 | 0.18 | -3.01 | 3.18x10^-3^ | 3.00x10^-2^ | January vs. July |
|  | -0.59 | 0.19 | -3.13 | 2.15x10^-3^ | 2.24x10^-2^ | January vs. May |
|  | 0.79 | 0.19 | 4.08 | 7.89x10^-5^ | 9.01x10^-4^ | March vs. July |
|  | 0.57 | 0.20 | 2.85 | 5.13x10^-3^ | 4.32x10^-2^ | October vs. May |
| Alphaproteobacteria | -0.51 | 0.15 | -3.54 | 5.56x10^-4^ | 6.34x10^-3^ | January vs. May |
|  | 0.63 | 0.17 | 3.62 | 4.16x10^-4^ | 5.16x10^-3^ | October vs. May |
| Armatimonadia | -0.58 | 0.19 | -3.08 | 2.53x10^-3^ | 2.64x10^-2^ | January vs. May |
|  | 0.69 | 0.22 | 3.16 | 1.95x10^-3^ | 2.23x10^-2^ | October vs. May |
| Bacteroidia | -0.76 | 0.22 | -3.42 | 8.33x10^-4^ | 9.50x10^-3^ | January vs. May |
|  | 0.89 | 0.23 | 3.88 | 1.63x10^-4^ | 2.02x10^-3^ | October vs. May |
| Berkelbacteria | 0.88 | 0.21 | 4.24 | 7.77x10^-5^ | 9.65x10^-4^ | October vs. July |
|  | 0.69 | 0.22 | 3.06 | 3.27x10^-3^ | 3.41x10^-2^ | October vs. March |
|  | 0.77 | 0.21 | 3.67 | 5.14x10^-4^ | 5.87x10^-3^ | October vs. May |
| Chthonomonadetes | 0.73 | 0.23 | 3.18 | 2.00x10^-3^ | 2.49x10^-2^ | October vs. May |
| Fimbriimonadia | -0.90 | 0.30 | -3.03 | 3.14x10^-3^ | 3.58x10^-2^ | January vs. July |
|  | 0.75 | 0.23 | 3.33 | 1.23x10^-3^ | 1.53x10^-2^ | October vs. July |
| Kapabacteria | -1.26 | 0.28 | -4.42 | 2.34x10^-5^ | 2.43x10^-4^ | January vs. July |
|  | 0.90 | 0.23 | 3.93 | 1.52x10^-4^ | 1.43x10^-3^ | March vs. July |
|  | 1.65 | 0.21 | 7.86 | 2.93x10^-12^ | 3.64x10^-11^ | October vs. July |
|  | 1.17 | 0.22 | 5.30 | 6.06x10^-7^ | 6.92x10^-6^ | October vs. May |
| Longimicrobia | -0.58 | 0.19 | -3.04 | 2.87x10^-3^ | 3.57x10^-2^ | January vs. May |
| Myxococcia | 0.66 | 0.20 | 3.33 | 1.15x10^-3^ | 1.43x10^-2^ | October vs. May |
| Oligoflexia | -0.68 | 0.18 | -3.74 | 2.78x10^-4^ | 2.90x10^-3^ | January vs. July |
|  | -0.91 | 0.22 | -4.11 | 7.12x10^-5^ | 8.84x10^-4^ | January vs. May |
|  | 0.76 | 0.21 | 3.57 | 5.01x10^-4^ | 4.72x10^-3^ | October vs. July |
|  | 0.99 | 0.25 | 4.00 | 1.06x10^-4^ | 1.21x10^-3^ | October vs. May |
| Phycisphaerae | -0.60 | 0.15 | -3.88 | 1.64x10^-4^ | 1.87x10^-3^ | January vs. May |
|  | 0.56 | 0.18 | 3.19 | 1.76x10^-3^ | 1.66x10^-2^ | October vs. July |
|  | 0.81 | 0.19 | 4.24 | 4.16x10^-5^ | 5.17x10^-4^ | October vs. May |
|  | -0.45 | 0.15 | -2.96 | 3.64x10^-3^ | 4.52x10^-2^ | January vs. May |
| Polyangia | -0.84 | 0.20 | -4.14 | 6.35x10^-5^ | 5.97x10^-4^ | January vs. July |
|  | -0.98 | 0.23 | -4.25 | 4.12x10^-5^ | 4.70x10^-4^ | January vs. May |
|  | 0.70 | 0.24 | 2.89 | 4.57x10^-3^ | 3.39x10^-2^ | March vs. July |
|  | 0.96 | 0.23 | 4.17 | 5.58x10^-5^ | 5.81x10^-4^ | October vs. July |
|  | 1.11 | 0.26 | 4.32 | 3.11x10^-5^ | 3.86x10^-4^ | October vs. May |
| Saccharimonadia | 0.73 | 0.25 | 2.99 | 3.58x10^-3^ | 4.45x10^-2^ | October vs. July |
|  | 0.77 | 0.21 | 3.60 | 5.29x10^-4^ | 6.56x10^-3^ | October vs. May |
| vadinHA49 | 0.96 | 0.21 | 4.48 | 2.16x10^-5^ | 2.46x10^-4^ | October vs. July |
|  | 1.12 | 0.23 | 4.82 | 5.77x10^-6^ | 7.16x10^-5^ | October vs. May |

Supplementary Table 12. Fungal orders that are differentially abundant across months and passed sensitivity analyses. “lfc” is the natural log-fold change, “se” is the standard error, and “W” is the test statistic.

| Order | lfc | se | W | p value | p adj. | Month |
| --- | --- | --- | --- | --- | --- | --- |
| Dothideales | -1.353 | 0.427 | -3.171 | 1.90x10^-3^ | **3.73x10^-2^** | January vs. July |
| Lichenostigmatales | -1.423 | 0.460 | -3.095 | 2.42x10^-3^ | **4.75x10^-2^** | January vs. July |
| Myriangiales | -1.777 | 0.548 | -3.242 | 1.93x10^-3^ | **3.79x10^-2^** | October vs. March |
| Rhizophydiales | 1.736 | 0.501 | 3.461 | 1.03x10^-3^ | **2.02x10^-2^** | October vs. May |
|  | -1.639 | 0.506 | -3.242 | 1.98x10^-3^ | **3.70x10^-2^** | May vs. March |
|  | -1.737 | 0.549 | -3.165 | 2.49x10^-3^ | **4.39x10^-2^** | January vs. May |

Supplementary Table 13. Mean biomass of fungi and bacteria by month. Italicized rows are multi-month means. OctJanMar:MayJul is the ratio of the mean of October, January, and March to May and July.

|  | Mean Fungal Biomass Percentage | Mean Bacterial Biomass Percentage |
| --- | --- | --- |
| October | 6.19 | 28.35 |
| January | 6.93 | 26.94 |
| March | 7.17 | 25.75 |
| May | 0.70 | 7.70 |
| July | 0.62 | 7.45 |
| *Oct/Jan/Mar Mean* | *6.77* | *27.01* |
| *May/Jul Mean* | *0.66* | *7.57* |
| OctJanMar:MayJul | **10.32** | **3.57** |

Supplementary Table 14. Average monthly temperature, average monthly humidity, and precipitation totals averaged across the three vegetation types. Sampled months are shown in bold. Data from J. Anderson, 2023abc.

| Month | Average Total Precipitation (mm) | Average Monthly Humidity (%) | Average Monthly Temp (C) |
| --- | --- | --- | --- |
| 22-Oct | **60.37** | **61.88** | **15.16** |
| 22-Nov | 0 | 43.74 | 7.53 |
| 22-Dec | 16.43 | 59.96 | 5.62 |
| 23-Jan | **10.08** | **50.57** | **5.53** |
| 23-Feb | 2.54 | 43.05 | 6.32 |
| 23-Mar | **5.93** | **34.73** | **11.69** |
| 23-Apr | 0 | 20.21 | 16.91 |
| 23-May | **30.05** | **29.18** | **21.64** |
| 23-Jun | 0 | 19.84 | 26.26 |
| 23-Jul | **8.55** | **28.28** | **30.56** |
